# Eriodictyol Attenuates Myocardial Ischemia-Reperfusion Injury through the Activation of JAK2

**DOI:** 10.3389/fphar.2018.00033

**Published:** 2018-01-30

**Authors:** Defang Li, Ning Lu, Jichun Han, Xiaoyu Chen, Wenjin Hao, Wenjuan Xu, Xiaona Liu, Lei Ye, Qiusheng Zheng

**Affiliations:** ^1^School of Integrated Traditional Chinese and Western Medicine, Binzhou Medical University, Yantai, China; ^2^Key Laboratory of Xinjiang Endemic Phytomedicine Resources, Ministry of Education, School of Pharmacy, Shihezi University, Shihezi, China; ^3^State Key Laboratory of Natural Medicines, China Pharmaceutical University, Nanjing, China

**Keywords:** eriodictyol, myocardial ischemia-reperfusion, apoptosis, inflammation, JAK2

## Abstract

Myocardial ischemia-reperfusion (I/R) injury remains the leading risk factor of disability and mortality worldwide. In this study, the myocardial protective effect of eriodictyol (EDT) and the underlying mechanism in an *ex vivo* model of global myocardial I/R was investigated. After treatment with different concentrations of EDT, the decreased hemodynamic parameters induced by myocardial I/R injury were significantly attenuated by EDT. The elevated levels of IL-6, CRP, IL-8, and TNF-α were effectively reduced by EDT treatment. EDT also remarkably suppressed the levels of Bax and cleaved Caspase-3, and up-regulated the level of Bcl-2 in cardiac tissues from EDT-treated groups. Further studies showed that EDT could increase the levels of p-JAK2 and p-STAT3 in cardiac tissues. Meanwhile, treatment of AG490, a specific inhibitor of JAK2, abolished the protective effect of EDT on hemodynamic parameters, myocardial inflammation and myocardial cell apoptosis induced by I/R injury. These results demonstrated that EDT could protect against myocardial I/R injury through the activation of JAK2, providing a potential treatment with EDT during myocardial I/R injury.

## Introduction

Myocardial ischemia, caused by a decrease of blood supply to cardiac tissue, remains the leading risk factor of disability and mortality worldwide (Zhao et al., 2016). Although reperfusion is a useful treatment to restore the blood supply, reperfusion itself leads to the abnormal metabolism of energy and subsequently cause tissue damage, which is called ischemia-reperfusion (I/R) injury. Myocardial I/R injury is the cardiac tissue damage led by the recovery of blood flow after heart ischemia, which could be caused by many clinical setting, e.g., coronary bypass surgery, thrombolytic therapy, percutaneous transluminal coronary angioplasty, cardiopulmonary resuscitation, cardiopulmonary bypass or organ transplantation (Yu et al., 2015; Li et al., 2017). Myocardial I/R injury is known to result in the acute inflammatory response, including neutrophil infiltration and cytokine release, and myocardial cell apoptosis. It is reported that multiple cytokines contribute to this inflammatory reaction, such as interleukin (IL)-6, C-reactive protein (CRP), IL-8 and tumor necrosis factor (TNF)-α (Wei et al., 2013). One study demonstrated that anti-IL-6 or anti-TNF-α could attenuate the myocardial injury induced by I/R (Gurevitch et al., 1997). In addition, myocardial cell apoptosis is also considered to be one of primary pathophysiological mechanisms of myocardial I/R injury (Yue et al., 2017). So, it is of great importance to attenuate the inflammatory response and myocardial cell apoptosis during myocardial I/R, enhancing the quality of life.

The Janus kinase (JAK)/signal transducer and activator of transcription (STAT) signaling, transducing cellular signals from the plasma membrane to the nucleus, has been proven to mediate cardio-protection against myocardial I/R injury. Until now, four JAKs molecules (JAK1, JAK2, JAK3, and TYK2) and seven STATs molecules (STAT1, STAT2, STAT3, STAT4, STAT5a, STAT5b, and STAT6) have been discovered (Horvath, 2000). Especially, JAK2/STAT3 signaling has been reported to protect against myocardial I/R injury by several compounds (Zhao et al., 2016; Li et al., 2017). As previous study has shown that scutellarin remarkably inhibited I/R injury-induced pro-inflammatory cytokines (e.g., IL-6, IL-8, and TNF-α) release, oxidative response and cardiomyocyte apoptosis by enhancing JAK2/STAT3 pro-survival signaling (Wang et al., 2016). In another study, cilostazol treatment significantly suppresses myocardial cell apoptosis through the activation of JAK2/STAT3 pathway during myocardial I/R injury, and the inhibitor AG490 can reverse the increase of Bcl-2 level and the decrease of Bax and cleaved caspase-3 levels induced by cilostazol (Li et al., 2017). Therefore, the activation of JAK2/STAT3 signaling could decrease the inflammatory response and myocardial cell apoptosis to protect against myocardial I/R injury.

Eriodictyol [2-(3,4-dihydroxyphenyl)-5,7-dihydroxy-2,3-dihydrochromen-4-one, EDT], a bitter- masking flavanone, is commonly present in fruits and vegetables, especially citrus such as lemon (Yanez et al., 2008). Recent studies show that EDT possesses anti-inflammatory and antioxidant properties. It is reported that EDT not only significantly inhibits lipopolysaccharide (LPS)-induced nitric oxide production in murine macrophage cell line RAW 264.7 (Zhang et al., 2017), but also down-regulated the increased expression of inflammatory genes (IL-1β, IL-6, IL-8, IL-10, and TNF-α) induced by LPS in human monocytic leukemia cell line THP-1 (Chanput et al., 2016). EDT also protects neurons against β-amyloid 25–35 peptide-induced oxidative cell death in primary cultured neurons by activation of Nrf2/ARE signaling pathway (Jing et al., 2015). Other study shows that EDT suppresses the content of malondialdehyde (MDA), reactive oxygen species (ROS), as well as the production of TNF-α, and IL-1β in cisplatin (CP)-induced kidney injury, implying that EDT protects against CP-induced kidney injury through its antioxidant and anti-inflammatory effects (Li et al., 2016). A recent study reveals that EDT prevents reduced infarct area, neuronal death and subsequently improves neurological and memory deficits during brain ischemia (Ferreira Ede et al., 2016). However, the effects of EDT on myocardial I/R injury and the exact mechanism remains unclear.

In this study, we first evaluated the effects of EDT on the heart hemodynamic parameters, myocardial infarct size, proinflammatory cytokines and myocardial cell apoptosis in *ex vivo* model of global myocardial I/R. Next, the apoptosis-related proteins and the activation of JAK2 and STAT3 in the cardiac tissues were examined by Western blot. Based on these results, we employed the special inhibitor of JAK2 in this I/R model, and then re-evaluated the heart Hemodynamic parameters, myocardial infarct size, proinflammatory cytokines and myocardial cell apoptosis to investigate the role of JAK2 activation in the myocardial protection of EDT.

## Materials and Methods

### Materials and Reagents

Eriodictyol (EDT) (≥95.0%, Cat No. 89061), AG 490 (ak-2 protein tyrosine kinase inhibitor, Cat No. T3434) and 2,3,5-Triphenyltetrazolium chloride (TTC, ≥95.0%, Cat No. T8877), were purchased from Sigma-Aldrich (Shanghai, China). Lactate dehydrogenase (LDH) Cytotoxicity Assay Kit (Cat No. C0017), Rat IL-6 Enzyme-Linked ImmunoSorbent Assay (ELISA) Kit (Cat No. PI328), Rat TNF-α ELISA Kit (Cat No. PT516) and Colorimetric TUNEL Apoptosis Assay Kit (Cat No. C1098) were purchased from Beyotime Biotechnology (Shanghai, China). Creatine Kinase Activity Assay Kit (Cat No. BC1140) Rat CRP EasyTest^TM^ ELISA kit (Cat No. 1030026) were purchased from Beijing Solarbio Science & Technology Co., Ltd., Rat IL-8 ELISA Kit (Cat No. BP-E30583) was purchased from Shanghai Langton Biotechnology Co., Ltd., Anti-Bax antibody (Cat No. sc-7480), Anti-Bcl-2 antibody (Cat No. sc-7382), Anti-Caspase-3 antibody (Cat No. sc-56053), Anti-JAK2 antibody (Cat No. sc-390539), Anti-STAT3 antibody (Cat No. sc-8019), Anti-p-STAT3 antibody (Cat No. sc-8059) and anti-β-actin antibody (Cat No. sc-47778) were all purchased from Santa Cruz Biotechnology. Anti-p-JAK2 antibody (Cat No. ab32101) was purchased from Abcam (Shanghai, China).

### Animals and Myocardial I/R Model

Ninety male Sprague Dawley (SD) rats, 250–300 g, were obtained from Xinjiang Medicine University Medical Laboratory Animal Center [License No. SCXK (xin) 2016-0016]. The rats were kept in the animal room at Shihezi University in accord with a commercial standard rat diet and water *ad libitum*. The room was maintained at a constant temperature of 25°C relative humidity of 70%, and a 12 h light/12 h dark cycle. Then, the *ex vivo* model of global myocardial I/R was established according our previous study (Han et al., 2017). Briefly, the rats were anesthetized with an intraperitoneal injection (i.p.) of chloral hydrate (330 mg/kg). And the rats were administered intraperitoneally with 250 U⋅kg^-1^ of heparin to prevent coagulation of the blood before the operation. Subsequently, the hearts were excised quickly by thoracic surgery from rats and mounted on Langendorff’s heart perfusion apparatus. The mounted hearts were perfused with a Krebs–Henseleit (K–H) buffer (120 mM NaCl, 1.2 mM MgSO_4_, 1.2 mM CaCl_2_, 1.2 mM KH_2_PO_4_, 11 mM glucose and 25 mM sodium acetate, pH 7.4), equilibrated with a gas mixture comprised of 5% CO_2_ and 95% O_2_ at 37°C, and then incubated in a water-jacketed organ chamber at 37°C. After 15 min of perfusion to stabilize the hearts, the hearts were subjected to 15 min of perfusion, 15 min of zero-flow global ischemia and subsequently 45 min of reperfusion. All the experimental protocols were approved by the Committees of Animal Ethics and Experimental Safety of Shihezi University are conform with the NIH guidelines for the care and use of laboratory animals.

### Study Groups and Experimental Design

To evaluate the protective effects of EDT on myocardial I/R injury and to confirm the role of JAK2 activation during the myocardial protection of EDT, two experimental studies were designed in the present study. For study 1: Forty rats were randomly subdivided into five groups, including (1) Control group (Control): the hearts were excised from normal rats and were stabilized for 15 min and subsequently perfused for 75 min, (2) myocardial I/R group (I/R): the hearts were excised from normal rats and were subjected to 15 min of perfusion, 15 min of zero-flow global ischemia and subsequently 45 min of reperfusion after 15 min of stabilization, (3) I/R+EDT-5 treatment group (I/R+EDT-5): the rats were treated with EDT (5 mg⋅kg^-1^⋅d^-1^, i.p., 3 days before the operation), then the hearts were excised from the rats and were administrated with a K–H buffer containing EDT (5 mg/L) for 15 min, 15 min of zero-flow global ischemia and subsequently 45 min of reperfusion after 15 min of stabilization, (4) I/R+EDT-10 treatment group (I/R+EDT-10): the rats were treated with EDT (10 mg⋅kg^-1^⋅d^-1^, i.p., 3 days before the operation), then the hearts were excised from the rats and were administrated with a K–H buffer containing EDT (10 mg/L) for 15 min, 15 min of zero-flow global ischemia and subsequently 45 min of reperfusion after 15 min of stabilization, (5) I/R+EDT-20 treatment group (I/R+EDT-20): the rats were treated with EDT (20 mg⋅kg^-1^⋅d^-1^, i.p., 3 days before the operation), then the hearts were excised from the rats and were administrated with a K–H buffer containing EDT (20 mg/L) for 15 min, 15 min of zero-flow global ischemia and subsequently 45 min of reperfusion after 15 min of stabilization.

For study 2: Thirty-two rats were randomly subdivided into 4 groups, including (1) myocardial I/R group (I/R): the hearts were excised from normal rats and were subjected to 15 min of perfusion, 15 min of zero-flow global ischemia and subsequently 45 min of reperfusion after 15 min of stabilization, (2) I/R+EDT treatment group (I/R+EDT): the rats were treated with EDT (20 mg⋅kg^-1^⋅d^-1^, i.p., 3 days before the operation), then the hearts were excised from the rats and were administrated with a K–H buffer containing EDT (20 mg/L) for 15 min, 15 min of zero-flow global ischemia and subsequently 45 min of reperfusion after 15 min of stabilization, (3) I/R+EDT+AG490 treatment group (I/R+EDT+AG490): the rats were treated with EDT (20 mg⋅kg^-1^⋅d^-1^, i.p., 3 days before the operation) and AG490 (5 mg⋅kg^-1^⋅d^-1^, i.p., 3 days before the operation), then the hearts were excised from the rats and were simultaneously administrated with a K–H buffer containing EDT (20 mg/L) and AG490 (5 mg/L) for 15 min, 15 min of zero-flow global ischemia and subsequently 45 min of reperfusion after 15 min of stabilization, (4) I/R+AG490 treatment group (I/R +AG490): the rats were treated with AG490 (5 mg⋅kg^-1^⋅d^-1^, i.p., 3 days before the operation), then the hearts were excised from the rats and were administrated with a K–H buffer containing AG490 (5 mg/L) for 15 min, 15 min of zero-flow global ischemia and subsequently 45 min of reperfusion after 15 min of stabilization.

### Hemodynamic Parameters Measurement

After the perfusion of the isolated rat hearts in Langendorff’s apparatus, a water-filled latex balloon coupled to a pressure transducer (Statham) was inserted into the left ventricular cavity via the left auricle for pressure recording. The PowerLab data acquisition (DAQ) device was employed to continuously monitor the hemodynamic parameters, including the left ventricular end-diastolic pressure (LVEDP), left ventricular systolic pressure (LVSP), left ventricular developed pressure (LVDP, LVDP = LVSP - LVEDP) and maximum rise/down velocity of the left intraventricular pressure (±dp/dt_max_). Meanwhile, an ultrasonic flowmeter (model T106) was used to determine the coronary flow (CF). The recorded data were analyzed by a 4S AD Instruments biology polygraph (PowerLab, Australia).

### Determination of LDH and CK Activities

The K–H buffers were collected 10 min before the end of reperfusion. The collected solutions were used to determine the activities of LDH and CK in a blinded manner as described before (Zhao et al., 2016). All the experimental procedures were performed according to the manufacturer’s instructions.

### Myocardial Infarct Size Measurement

After 45 min of reperfusion, the hearts were collected and washed with ice-cold phosphate buffered saline (PBS). Then the hearts were stored and frozen at -80°C for 5 min. The frozen hearts sectioned into five pieces perpendicularly along the long axis from apex to base. Subsequently, the heart slices were incubated with 1% TTC buffer (pH 7.4) for 15 min at 37°C. After the incubation, the heart slices were fixed using a 4% formaldehyde solution. Finally, the fixed heart slices were photographed by a digital camera. The areas of white-unstained necrotic tissues and red-stained viable tissues were measured by Image-Pro Plus 7.0 (Media Cybernetics, United States) and used to calculate the infarct size percentage of myocardium. The concrete formula is as follows: infarct volume percentage = (Infarct volume/Total volume of slices) × 100.

### Pro-inflammatory Cytokines Examination

After the reperfusion, cardiac tissues were harvested and homogenized (100 mg/mL) in ice-cold physiological saline. The homogenates were centrifuged at 3000 g and 4°C for 15 min. The supernatant was isolated and the protein concentration of the supernatant was measured by Pierce^TM^ BCA Protein Assay Kit (Thermo Fisher Scientific, Cat No. 23225). Then, the content of IL-6, CRP, IL-8, and TNF-α were examined using the commercial ELISA kits. All the experimental procedures were performed according to the manufacturer’s instructions.

### TUNEL Assay

The hearts were fixed by 4% paraformaldehyde in PBS solution (pH 7.4) at room temperature for 24 h. The fixed cardiac tissues were then embedded in paraffin and sliced into 4 μm sections. The slices were deparaffinized, rehydrated, and treated with protease K (10 mmol/L) for 15 min. After treatment, the slides were employed for TUNEL staining according to the manufacturer’s instructions as described in our previous study (Han et al., 2017).

### Western Blot Analysis

The RIPA buffer [20 mmol/L Tris-HCl (pH 7.4), 2 mmol/L EGTA, 150 mmol/L sodium chloride, 1% Triton-X 100, 0.5% sodium deoxycholate, 2 mmol/L sodium orthovanadate and 100 mmol/L sodium fluoride] containing a cocktail of protease inhibitors (Roche, West Sussex, United Kingdom) was used to extract proteins from rat hearts. The concentrations of the extracted proteins were quantified by Pierce^TM^ BCA Protein Assay Kit. Then the proteins were boiled for 5 min and subsequently separated by electrophoresis on a 12% SDS polyacrylamide gel. After separating, the proteins were transferred to PVDF membranes. The membranes were incubated with the primary antibodies against Bax, Bcl-2, Caspase-3, JAK2, p-JAK2, STAT3, p-STAT3, and β-actin overnight at 4°C. Then, the membranes were washed with TBST buffer and incubated with horseradish peroxidase-conjugated secondary antibody in TBST buffer at 37°C for 2 h. Finally, the membranes were washed and visualized using Pierce^TM^ ECL Plus Western Blotting Substrate (Thermo Fisher Scientific, Cat No. 32132), and the bands were scanned and quantified using Bio-Rad Gel Doc 2000 imaging system.

### Statistical Analysis

The data are presented as the mean ± standard deviation (SD). A two-tailed Student *t*-test for unpaired examinations or a one-way ANOVA followed by the Bonferroni *post hoc* test for multiple comparisons was performed for statistical analyses. *P* < 0.05 was considered statistically significant.

## Results

### EDT Treatment Significantly Attenuates I/R-Induced Myocardial Injury

**FIGURE 1:**
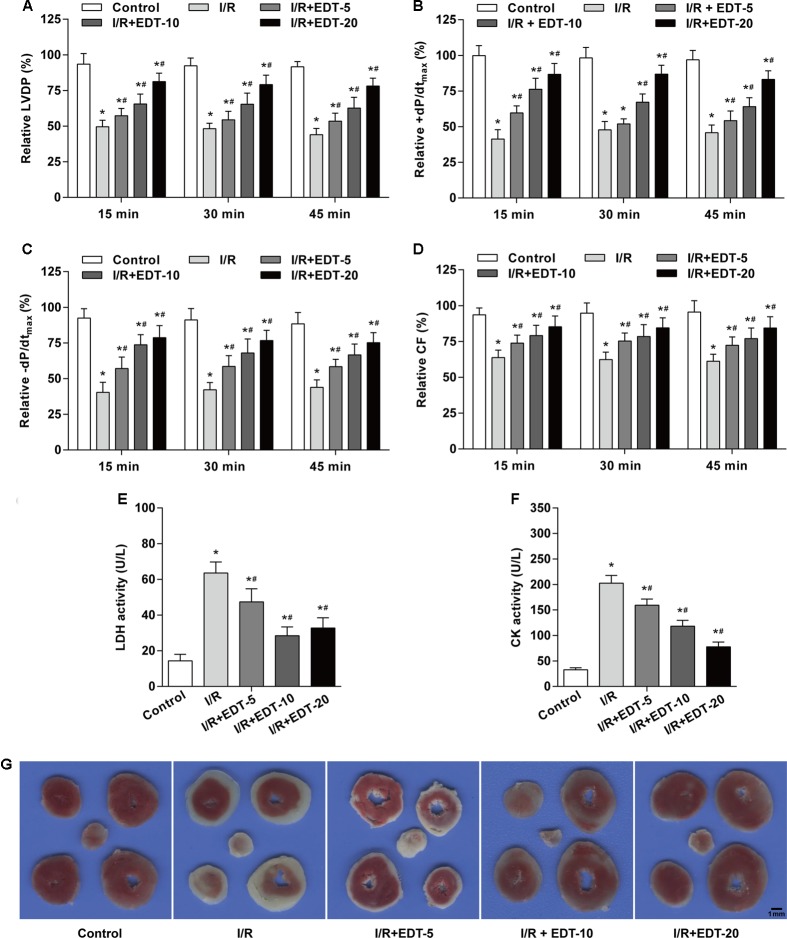
Eriodictyol (EDT) treatment rescues myocardial injury in an *ex vivo* model of global myocardial I/R. The effect of EDT on LVDP **(A)**, +dP/dt_max_ **(B)**, –dP/dt_max_ **(C)**, and CF **(D)** in rat hearts were measured using PowerLab data acquisition device. The values of these parameters were normalized to the values of the recorded data before 15 min of zero-flow global ischemia. The levels of LDH **(E)** and CK **(F)** in K–H buffer were determined by commercial kits. **(G)** The infarct size was examined by TTC staining after EDT treatment. Scale bar: 1 mm. *n* = 8 for each group. All data are the mean ± SD. *^∗^P* < 0.05 compared with control group, ^#^*P* < 0.05 compared with I/R group.

In order to confirm the myocardial protective effect of EDT, the hemodynamic parameters were examined in heart after pretreatment and treatment with different concentrations of EDT. Compared with the control group, the Hemodynamic parameters (LVDP, +dP/dt_max_, -dP/dt_max_, and CF) were remarkably decreased in I/R group (**Figures 1A–D**). After pretreatment and treatment with EDT, we found that the decreased Hemodynamic parameters induced by myocardial I/R were significantly attenuated in M-EDT and H-EDT treatment groups (**Figures 1A–D**). Moreover, the LDH and CK levels in K–H buffer were increased in I/R group when compared with the control group, and EDT treatment effectively improved the changes induced by I/R injury (**Figures 1E,F**). Furthermore, we evaluated the infracted myocardium using TTC staining. The myocardial infarct size was markedly decreased by treatment with EDT when compared with the I/R group (**Figure 1G**).

### EDT Treatment Decreases the Pro-inflammatory Factors in the Myocardium

**FIGURE 2:**
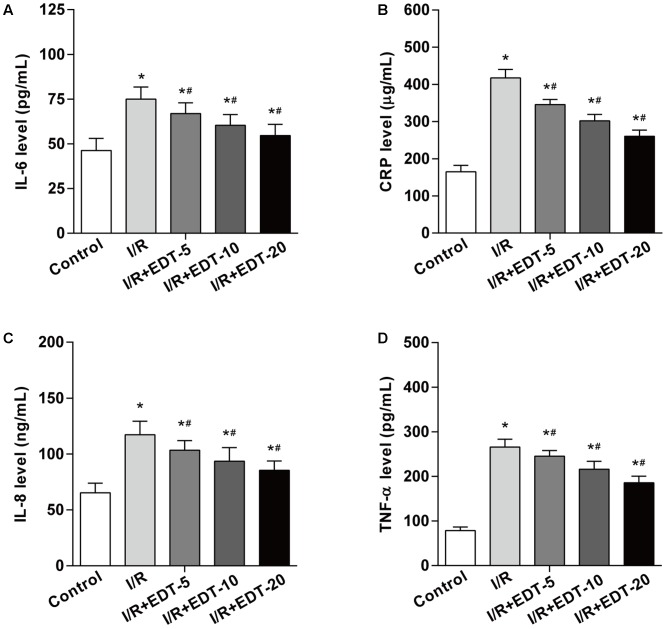
Eriodictyol treatment suppresses the elevated levels of IL-6, CRP, IL-8, and TNF-α induced by I/R. The effect of EDT on IL-6 **(A)**, CRP **(B)**, IL-8 **(C)**, and TNF-α **(D)** levels in cardiac tissues were measured by ELISA kits. *n* = 8 for each group. All data are the mean ± SD. *^∗^P* < 0.05 compared with control group, ^#^*P* < 0.05 compared with I/R group.

To determine whether EDT treatment can interfere with inflammatory response induced by I/R in the myocardium, the cardiac tissue obtained from each group were examined for the levels of IL-6, CRP, IL-8, and TNF-α. The levels of IL-6, CRP, IL-8, and TNF-α in cardiac tissue were significantly upregulated in I/R-injured myocardium when compared to those in control group. Notably, the elevated levels of IL-6, CRP, IL-8, and TNF-α were effectively reduced by EDT treatment in a dose-dependent manner (**Figures 2A–D**).

### EDT Treatment Reduces the Myocardium Cell Apoptosis Induced by I/R Injury

**FIGURE 3:**
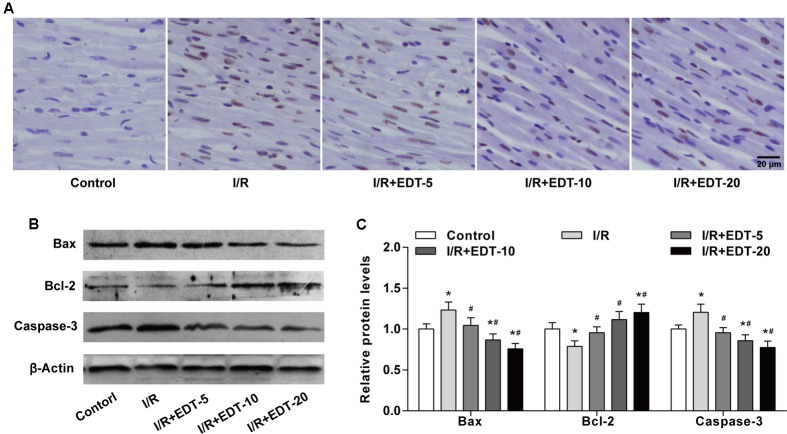
Eriodictyol treatment inhibits the cardiomyocyte apoptosis induced by I/R injury. **(A)** The cardiomyocyte apoptosis was examined by TUNEL assay. Brown staining of the nucleus indicates cell apoptosis. Scale bar: 20 μm. **(B)** The protein levels of Bax, Bcl-2 and cleaved Caspase-3 in cardiac tissues were measured by western blot. **(C)** Statistical analysis of Bax, Bcl-2 and cleaved Caspase-3 protein levels in cardiac tissues. *n* = 4 per group. The protein ratios were normalized to the values of the control group. All data are the mean ± SD. *^∗^P* < 0.05 compared with control group, ^#^*P* < 0.05 compared with I/R group.

Considering myocardium cell apoptosis is one important process in the pathogenesis of myocardial I/R injury, we next evaluated the effect of EDT on cell apoptosis induced by I/R in the myocardium. Compared with the control group, there was a significant higher myocardium apoptotic cell rate in I/R group. However, the EDT treatment significantly decreased the elevated cell apoptotic rate induced by myocardial I/R in the myocardium (**Figure 3A** and Supplementary Figure 1). Meanwhile, the pro-apoptotic proteins Bax and Caspase-3 were up-regulated and the anti-apoptotic protein Bcl-2 was down-regulated in I/R group when compared with the control group (**Figures 3B,C**). After treatment with EDT, the levels of Bax and Caspase-3 were significantly decreased and the Bcl-2 level was increased in the cardiac tissue when compared to those in the I/R group (**Figures 3B,C**).

### EDT Treatment Increases the Activation of JAK2

**FIGURE 4:**
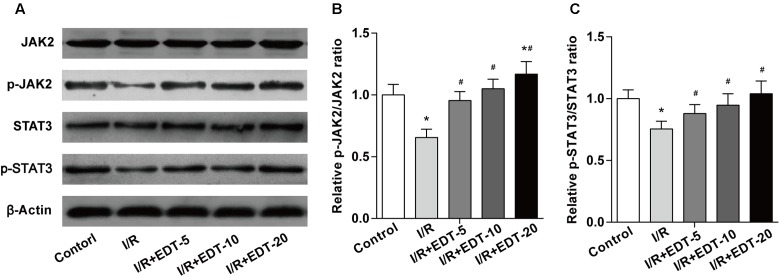
Eriodictyol treatment elevates the JAK2 activation. **(A)** The protein levels of JAK2, p-JAK2, STAT3, and p-STAT3 in cardiac tissues were measured by western blot. Statistical analysis of p-JAK2/JAK2 ratio **(B)** and p-STAT3/STAT3 ratio **(C)** in cardiac tissues. *n* = 4 per group. The protein ratios were normalized to the values of the control group. All data are the mean ± SD. *^∗^P* < 0.05 compared with control group, ^#^*P* < 0.05 compared with I/R group.

To further explore the molecular mechanism of EDT’s cardio-protective action, we examined the protein levels and the protein phosphorylation levels of JAK2 and STAT3 in cardiac tissue. Western blot analysis showed that no obvious change of the levels of JAK2 and STAT3 was found between control group and I/R group or between I/R group and I/R+EDT groups. But the levels of p-JAK2 and p-STAT3 in EDT-treated groups were both significantly elevated in cardiac tissue when compared to those with the I/R group (**Figures 4A,B**). Meanwhile, the ratios of p-JAK2/JAK2 and p-STAT3/STAT3 were both increased in I/R+EDT groups when compared with I/R group (**Figure 4C**).

### AG490 Abolishes the Cardio-Protection of EDT in Myocardium I/R Injury

**FIGURE 5:**
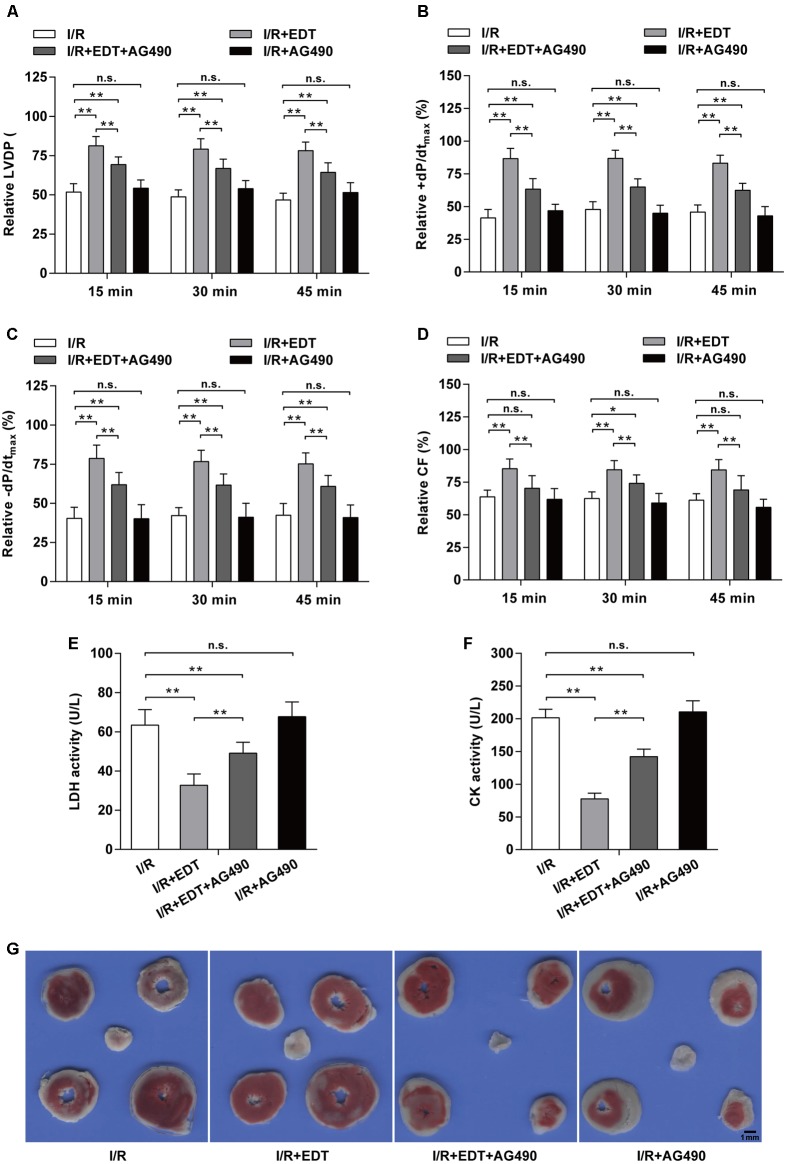
JAK2 specific inhibitor AG490 abolishes the protective effect of EDT in myocardium I/R injury. The values of LVDP **(A)**, +dP/dt_max_ **(B)**, -dP/dt_max_ **(C)**, and CF **(D)** in rat hearts were measured after co-treatment with EDT and AG490. The values of these parameters were normalized to the values of the recorded data before 15 min of zero-flow global ischemia. The levels of LDH **(E)** and CK **(F)** in K–H buffer were determined after co-treatment with EDT and AG490. **(G)** The infarct size was examined by TTC staining after co-treatment with EDT and AG490. Scale bar: 1 mm. *n* = 8 for each group. All data are the mean ± SD. *^∗^P* < 0.05, ^∗∗^*P* < 0.01.

To further confirm whether JAK2 activation can participate in the cardio-protection of EDT in I/R injury, we employed AG490 to inhibit the JAK2 activation and subsequently evaluated the influence of AG490 on the cardio-protection of EDT in rat heart I/R model. We found that the elevated Hemodynamic parameters (LVDP, +dP/dt_max_, -dP/dt_max_, and CF) induced by EDT were blocked by AG490 co-treatment (**Figures 5A–D**). AG490 alone (I/R+AG490 group) didn’t have a significant effect on those Hemodynamic parameters when compared with the I/R group (**Figures 5A–D**). Meanwhile, compared with I/R group, the decreased LDH and CK levels in I/R+EDT group were also obviously blocked by AG490 co-treatment (**Figures 5E,F**). Subsequently, the protective effect of EDT on myocardial infarct size was suppressed by AG490 co-treatment (**Figure 5G**).

### AG490 Abolishes the Effect of EDT on the Inflammatory Response and the Myocardium Cell Apoptosis Induced by I/R Injury

**FIGURE 6:**
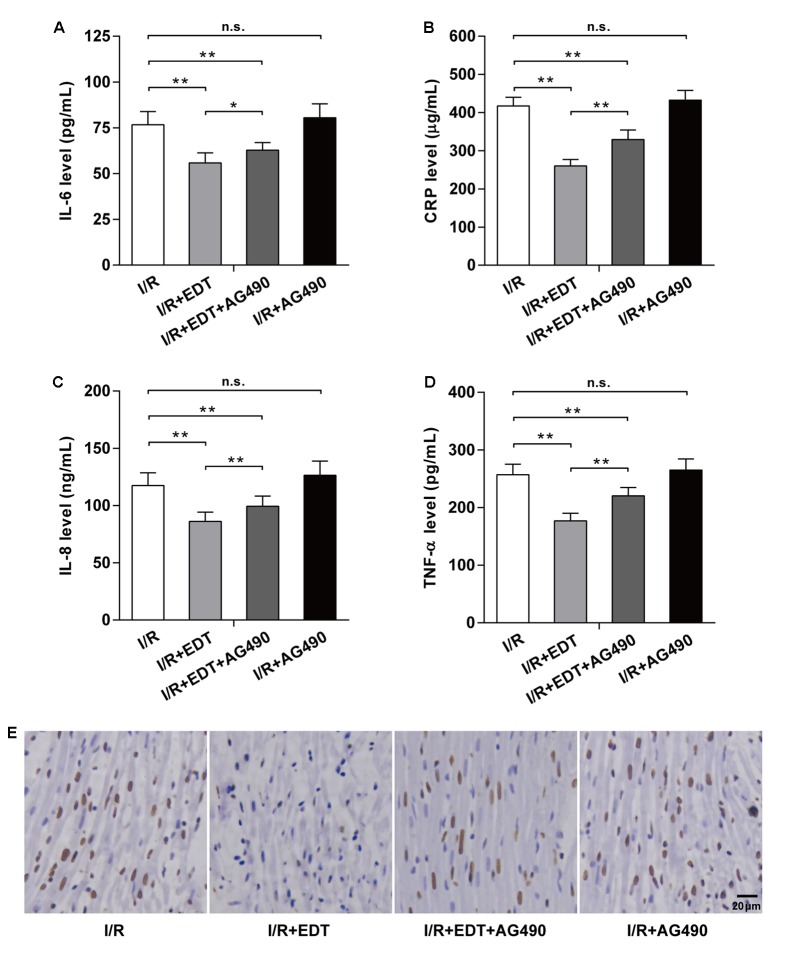
AG490 abolishes the effect of EDT on inflammatory response and cardiomyocyte apoptosis in global myocardial I/R. The effect of EDT on IL-6 **(A)**, CRP **(B)**, IL-8 **(C)**, and TNF-α **(D)** levels in cardiac tissues were measured after co-treatment with AG490. **(E)** The cardiomyocyte apoptosis in cardiac tissues was examined after co-treatment with EDT and AG490. Brown staining of the nucleus indicates cell apoptosis. Scale bar: 20 μm. *n* = 8 for each group. All data are the mean ± SD. ^∗^*P* < 0.05, ^∗∗^*P* < 0.01.

Compared with I/R group, the reductions of IL-6, CRP, IL-8, and TNF-α in I/R+EDT group were suppressed by AG490 co-treatment, but AG490 alone (I/R+AG490 group) didn’t have an obvious effect on those inflammatory factors when compared with the I/R group (**Figures 6A–D**). Moreover, the inhibition effect of EDT on myocardium cell apoptosis rate was attenuated by AG490 co-treatment, whereas no significant differences in the myocardium cell apoptosis rate were found between I/R group and I/R+AG490 group (**Figure 6E**).

### AG490 Abolishes the Effect of EDT on the Expression of Apoptosis-Related Proteins and the Activation of JAK2 in Myocardium I/R Injury

**FIGURE 7:**
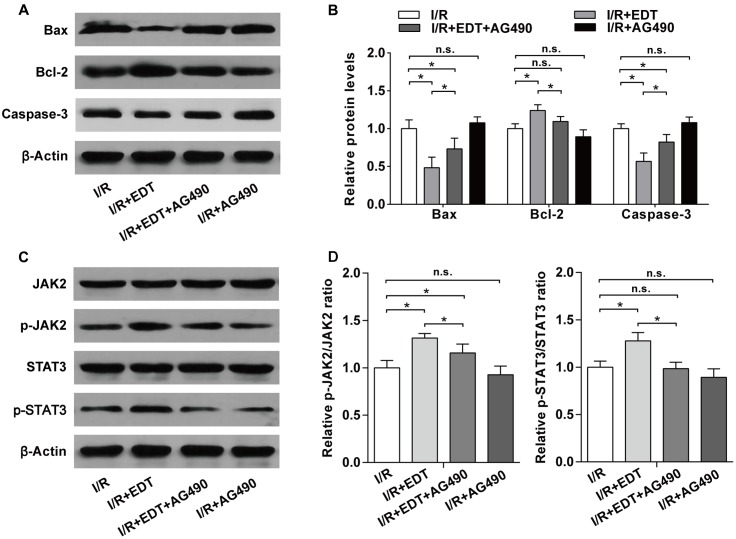
AG490 abolishes the effect of EDT on the expression of apoptosis-related proteins and the activation of JAK2 in myocardium I/R injury. **(A)** The protein levels of Bax, Bcl-2 and cleaved Caspase-3 in cardiac tissues were measured by western blot. **(B)** Statistical analysis of Bax, Bcl-2 and cleaved Caspase-3 protein levels in cardiac tissues from the indicated groups. **(C)** The protein levels of JAK2, p-JAK2, STAT3, and p-STAT3 in cardiac tissues were measured by western blot. **(D)** Statistical analysis of p-JAK2/JAK2 ratio and p-STAT3/STAT3 ratio in cardiac tissues from the indicated groups. *n* = 4 per group. The protein ratios were normalized to the values of the control group. All data are the mean ± SD. *^∗^P* < 0.05.

We further examined the influence of AG490 on the expression of apoptosis-related proteins and the activation of JAK2 in EDT-treated rat hearts. Compared with I/R group, the decreases of pro-apoptotic proteins (Bax and Caspase-3) and the increase of Bcl-2 induced by EDT were inhibited by AG490 co-treatment in I/R+EDT+AG490 group, but AG490 alone didn’t have a significant effect on these protein levels in AG490-treated group (**Figures 7A,B**). In addition, the increased ratios of p-JAK2/JAK2 and p-STAT3/STAT3 induced by EDT were also suppressed by AG490 co-treatment in I/R+EDT+AG490 group when compared with those in I/R+EDT group, while no change was found in AG490-treated group when compared with the I/R group (**Figures 7C,D**).

## Discussion

Until now, EDT has attracted more interesting as a therapeutic agent in many diseases, such as hypercholesterolemia, cerebral ischemia, cardiovascular diseases and neurodegenerative diseases (Jing et al., 2015; Lee et al., 2015; Ferreira Ede et al., 2016; Yamashita et al., 2016). In the present study, we utilized an *ex vivo* model to explore the protective effect of EDT against myocardial I/R-induced cardiac injury. We found that EDT markedly attenuated the decreased Hemodynamic parameters (LVDP, +dP/dt_max_, -dP/dt_max_, and CF), the increased myocardial infarct size, proinflammatory cytokine levels and myocardial cell apoptosis rate induced by myocardial I/R. Importantly, the JAK2 inhibitor AG490 attenuated the protective effect of EDT on myocardial I/R injury, indicating JAK2 activation could play a regulatory role in this process. This study could help us to understand the pharmacology and mechanism of EDT in the treatment of myocardial ischemia disease.

Inflammatory response is an important process during the pathogenesis of myocardial I/R injury. Many pro-inflammatory factors, e.g., IL-1β, IL-6, IL-8, IL-10, and TNF-α, are up-regulated and contribute to myocardial I/R injury. Yu et al. (2010) revealed that the decreasing expression levels of proinflammatory cytokines (IL-1β and TNF-α) are closely associated with the mechanism of ischemic preconditioning for myocardial I/R injury. The inhibition of IL-1β induced by caspase 1 reduces myocardial ischemic dysfunction in isolated human atrial myocardium (Pomerantz et al., 2001). Anderson et al. (2013) showed IL-6 and soluble IL-6 receptor are associated with acute myocardial infarction and cardiac injury. To further study the role of IL-6 in the temporal development of myocardial I/R injury, a closed-chest I/R model in IL-6-deficient mice was employed. The result demonstrated that IL-6 contributes to the development of infarct size in the early phase of myocardial I/R (Jong et al., 2016). TNF-α is another critical pro-inflammatory cytokine in myocardial I/R. Previous studies have found that TNF-α antagonism or inhibitor can ameliorate myocardial I/R injury in mice (Gao et al., 2015; Pei et al., 2015). Our previous study showed that rosmarinic acid can protects against myocardial I/R injury through down-regulating the levels of inflammatory cytokines (IL-6, CRP, and TNF-α) (Han et al., 2017). In this study, the data identified that EDT also can ameliorate myocardial I/R injury by modulating inflammatory response.

Apoptosis, also called programmed cell death, is another critical process during the pathogenesis of myocardial I/R injury. Although apoptosis has been recognized as a type of physiological phenomena during life development, elevated or insufficient apoptosis under some pathophysiologic conditions may lead to tissue injury and dysfunction (Chinda et al., 2014; Sun et al., 2016). It is reported that the number of myocardial apoptosis cell gradually increased in rat myocardial I/R model following ischemia and reperfusion time extension. Then, numerous cell apoptosis in cardiac tissue led to myocardial cell loss, thus affecting myocardial contraction and resulting in myocardial injury (Wu et al., 2013). Further study demonstrated that the apoptosis of myocardial cell participated in the occurrence and development of I/R injury. *In vitro* myocardial I/R model indicated that myocardial cells appeared apoptosis at 10 min after ischemia and reached peak at 30 min (Kuhla et al., 2008). *In vivo* myocardial I/R model revealed that sustained ischemia for 45 min followed by reperfusion for 1 h could lead to cell apoptosis (Liu et al., 2017). The number of myocardial apoptotic cell was increased following the reperfusion time extension (Liu et al., 2017). Previous studies have showed that EDT can protect against cell apoptosis and cell death in keratinocytes or PC12 cells (Lee et al., 2007; Lou et al., 2012). Consistent with these studies, we found that EDT significantly up-regulated the Bcl-2 level and down-regulated the Bax and cleaved caspase-3 levels, confirming the anti-apoptosis ability of EDT in myocardial I/R injury (Li et al., 2017).

Recent studies have confirmed that the JAK2/STAT3 signaling plays a pivotal role in many physiological processes, e.g., inflammation and apoptosis. It is reported that cilostazol decreases the inflammatory cytokines (IL-6, IL-1β, and TNF-α) by the activation of JAK2 and STAT3 during myocardial I/R injury. On the other hand, Jiang et al. (2015) have reported that the activation of JAK2/STAT3 is sufficient to protect against myocardial cell apoptosis induced by I/R, and AG490 abolish the protective effect of berberine on myocardial I/R injury (Zhao et al., 2016). In this study, we found that the phosphorylation levels of JAK2 and STAT3 were all remarkably activated by EDT. Based on these data, the specific JAK2 inhibitor AG490 was employed to test whether JAK2 activation can participate in the cardio-protection of EDT in myocardial I/R injury. The results showed that AG490 could significantly reversed the down-regulated levels of pro-inflammatory cytokines (IL-6, CRP, IL-8, and TNF-α) and the decreased myocardium cell apoptosis rate. The elevated Hemodynamic parameters (LVDP, +dP/dt_max_, -dP/dt_max_, and CF) and decreased LDH and CK levels in EDT-treated group were also suppressed by AG490, indicating that the activation of JAK2 was involved in the cardio-protection of EDT.

There are two limitations in the study should be acknowledged. First, we evaluated the protective effect of EDT on an *ex vivo* model of global myocardial I/R, but the protective effect or the long-term consequence of EDT on a *in vivo* myocardial I/R injury was not characterized. Second, we utilized only one JAK2 inhibitor (AG490) in this study. It is reported that AG490 and WP1066 are the two commonly used JAK2 inhibitors (Verstovsek et al., 2008; Zyuz’kov et al., 2017). However, WP1066 was recently found to block signal transducer and activator of transcription (STAT) and phosphoinositide-3-kinase pathways (Jing et al., 2015). Thus, we selected only AG490 for the present study.

Taken all the above findings, we speculate that EDT could suppress pro-inflammatory response and myocardial cell apoptosis through the activation of JAK2. These results may provide a potential treatment with EDT during myocardial I/R injury.

## Author Contributions

DL and QZ supervised the whole project. DL and NL performed the major research and wrote the manuscript in equal contribution. JH, XC, WH, WX, and XL provided the technical support. LY provided her professional expertise.

## Conflict of Interest Statement

The authors declare that the research was conducted in the absence of any commercial or financial relationships that could be construed as a potential conflict of interest.
