# Supplementary material for: Eriodictyol Attenuates Myocardial Ischemia-Reperfusion Injury through the Activation of JAK2
Source: Front Pharmacol. 2018 Jan 30;9:33. doi: 10.3389/fphar.2018.00033 (PMC5797583; doi:10.3389/fphar.2018.00033)
Supplement: Supplementary file 1 [file Data_Sheet_1.docx]

**Eriodictyol attenuates myocardial ischemia-reperfusion injury through the activation of JAK2**

**Supplementary figure 1**

**
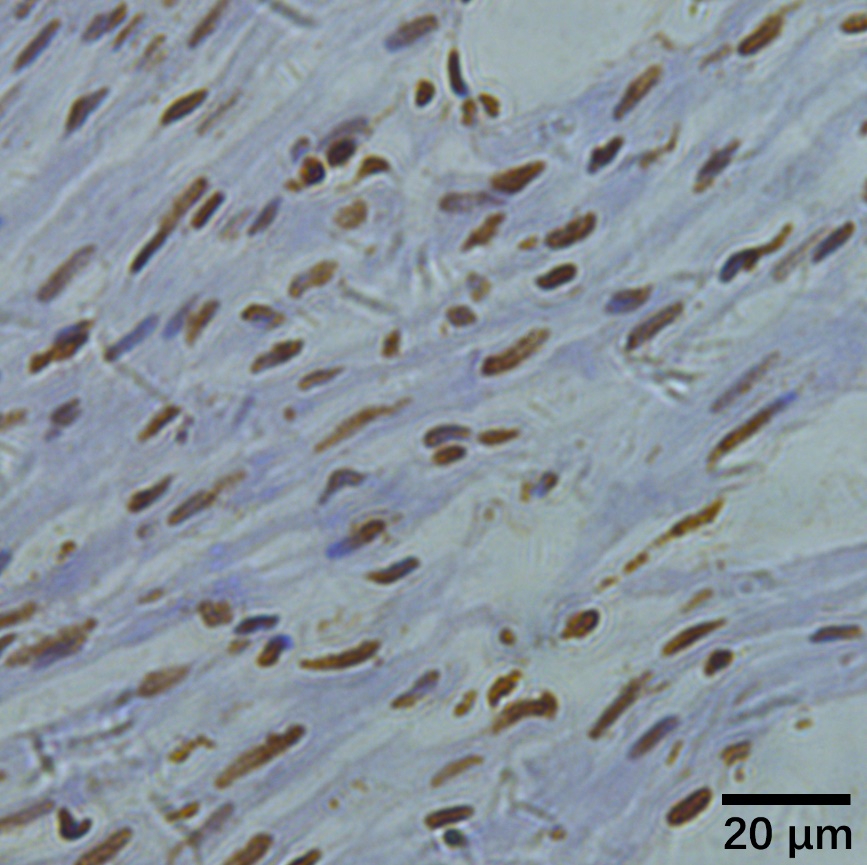
**

**Supplementary figure 1** The representative image of the positive control for TUNEL assay. Brown staining of the nucleus indicates cell apoptosis. Scale bar: 20 μm.
